# Reflectance confocal microscopy for plaque psoriasis therapeutic follow-up during an anti-interleukin-17A monoclonal antibody: an observational study

**DOI:** 10.1038/s41598-024-65902-8

**Published:** 2024-07-02

**Authors:** Qian Jiang, Zilu Qu, Bei Wang, Ruili Jiang, Yu Zhou, Li Wan, Liuqing Chen, Feng Hu

**Affiliations:** 1 Department of Dermatology, Traditional Chinese and Western Medicine Hospital of Wuhan, Tongji Medical College, Huazhong University of Science and Technology, Wuhan, 430022 China; 2 Department of Dermatology, Wuhan No.1 Hospital, Wuhan, 430022 China; 3 Hubei Province and Key Laboratory of Skin Infection and Immunity, Wuhan, 430022 China; 4 Clinical College of Traditional Chinese Medicine, Hubei University of Chinese Medicine, Wuhan, 430000 China; 5 Dermatology Hospital of Southern Medical University, Guangzhou, China. No. 2 Lu Jing Road, Yuexiu District, Guangzhou City, 510000 China

**Keywords:** Plaque psoriasis, Reflectance confocal microscopy, Non-invasive follow-up, Secukinumab treatment monitoring, Diseases, Medical research, Pathogenesis

## Abstract

Interleukin-17A therapeutic inhibitors are among the most effective treatment methods for moderate-to-severe plaque psoriasis (PP). Reflectance confocal microscopy is a non-invasive imaging technique already documented to be beneficial in evaluating the follow-up of PP under treatment with topical actives and phototherapy. This study aimed to assess the epidermal and dermal changes associated with psoriasis and its treatment with RCM during systemic secukinumab treatment in patients with moderate-to-severe PP. A pilot study was conducted to evaluate RCM as a non-invasive tool for monitoring secukinumab treatment in patients with PP. For patients receiving secukinumab treatment, lesional skin was selected for RCM imaging, which were recorded at all scheduled times. The RCM evaluation criteria were established based on the histopathological diagnostic criteria for psoriasis. The clinical severity of psoriasis was assessed utilizing the psoriasis area severity index. A total of 23 patients with PP were included in the study. Each patient received 300 mg of subcutaneous secukinumab as induction therapy at baseline and weeks 1–4, followed by maintenance therapy every four weeks. Microscopic confocal changes were observed during the treatment. The results identified early microscopic evidence of the anti-inflammatory activity of secukinumab, which was not detected during the clinical examination. RCM findings correlating with the PASI were used to observe the patient’s response to treatment and were identified as follows: acanthosis and parakeratosis, presence of epidermal and dermal inflammatory cells, presence of non-edge dermal papillae, and vascularization in the papillary dermis. This study is the first to demonstrate the use of RCM as an effective tool for non-invasive monitoring of secukinumab therapeutic response at a cellular level in a clinical or research setting. Early detection of RCM parameters associated with secukinumab activity may facilitate the identification of an early treatment response. RCM appears to be capable of providing practical and helpful information regarding follow-up in patients with PP undergoing secukinumab treatment. RCM may also provide novel perspectives on the subclinical evaluation of PP’s response to biological therapy.

## Introduction

Psoriasis is a complex, chronic immune-mediated skin disease with 0.6–4.8% worldwide prevalence. Plaque psoriasis (PP), the most common variant comprising over 80% of psoriasis cases, is characterized by erythematous scaly patches or plaques that frequently manifest on extensor surfaces. However, it may also affect the intertriginous areas, palms, soles, and nails^1–3^.

Secukinumab is a fully human monoclonal antibody that selectively neutralizes IL-17A, the cornerstone cytokine involved in the development of psoriasis^4^. By directly inhibiting IL-17A, secukinumab acts downstream of all possible alternative pathways of IL-17A release^5^. Secukinumab inhibits cytokines irrespective of the cell source, thus exerting an immunomodulatory effect in PP treatment^6^. Clinical cure of lesions is not the optimal time to discontinue biologics^7^. Assessment of the safety and efficacy of biological therapies for psoriasis and the basis for biologics reduction and discontinuation consistently relies on body surface area (BSA) and psoriasis area severity index (PASI)^3,8^. However, the actual implementation of these physical measures is limited by the possibility of subjective interpretation with interobserver variation^9^. Moreover, the propensity of psoriasis to migrate and reoccur presents significant challenges in treatment. Therefore, therapeutic monitoring and follow-up are essential. BSA and PASI are inadequate for revealing microscopic alterations during therapy and are less significant for predicting the long-term prognosis of psoriasis.

Reflectance confocal microscopy (RCM) has been increasingly used in recent years to investigate subclinical structural changes in psoriatic skin lesions^10–12^. This technique has demonstrated a strong correlation with histological findings and can quantify the dynamics of histological changes during therapy^13^. This study aimed to conduct an in vivo evaluation of the efficacy of RCM in the therapeutic follow-up of PP and disease progression during treatment with secukinumab based on the observation of detailed microscopic changes. Moreover, the data can provide insight into dynamic changes occurring in psoriasis lesions, including epidermal turnover, inflammatory status of the lesion, melanocyte activity, vascularization and alterations in the orientation of blood vessels. The identification of subclinical signs of early response to treatment was assessed for clinical application of the results. The RCM can be used in detailed monitoring of microscopic changes to evaluate the timing of patient drug reduction and discontinuation, thereby avoiding disease recurrence and over-treatment for the management of PP during biologic therapies.

## Materials and methods

### Patients

We included patients with psoriasis treated with secukinumab injection at the Department of Dermatology of Wuhan No.1 Hospital in 2022. This study was approved by the Research Ethics Committee of Wuhan No.1 Hospital and conformed to the Declaration of Helsinki. All patients provided written informed consent before inclusion.

The diagnosis was established through clinical observations and further validated by histological examination. Each patient exhibited moderate to severe psoriasis plaques and met all the clinical criteria for secukinumab treatment. For each patient, one or more stable psoriasis plaques showing a mild-to-moderate scaling degree, easily accessible for examination, and localized in three different body areas (arms, legs, and trunk) were selected. Lesions covered by thick scales or located in hairy areas were avoided. Patients who had undergone phototherapy and/or a history of sun exposure in the previous four weeks, pregnancy, smoking and/or alcohol consumption, and the presence of microangiopathic diseases, including diabetes and connective vascular diseases, were excluded.

### Study protocol

During this single-center observational study, RCM examinations were done on the centre of the target lesion before starting biological treatment (T0), after 1 weeks (T1), 2 weeks (T2), 3 weeks (T3), 4 weeks (T4) and 8 weeks (T5) of treatment. The PASI score was calculated throughout every observation for global disease assessment. Photographic images were acquired at baseline and every single study visit for clinical evaluation of the response to treatment.

### Secukinumab therapy

Patients undergoing secukinumab therapy were enroled in the study. It was selected because of its simple administration, proven efficacy in moderate to severe PP, and safety profile. Each patient received 300 mg of subcutaneous secukinumab at baseline (week 0) and at weeks 1–4 as induction therapy, followed by maintenance therapy every four weeks after that.

### Reflectance confocal microscopy imaging and analysis

Confocal imaging was conducted using the commercially accessible VivaScope 1500 System (Lucid Inc.). A more comprehensive exposition of the system has been previously published. The RCM imaging was executed according to a standardized protocol. Horizontal mapping was performed at various skin levels, and Viva BlockTM software was employed to generate mosaics of 16 images (0.5 × 0.5 mm) at the same skin level for descriptive analysis. Vertical mapping was performed by capturing images using the Vertical Viva StackTM software. Stacked images were captured starting at the initial nucleated epidermal layer and progressing deeper in 5-μm-steps until the epidermis-dermis junction or dermis was reached or until the image clarity deteriorated (180 μm maximum).

On the basis of optical histology of PP, RCM was used for the evaluation of the presence of following microscopic features, namely: parakeratosis, epidermal and dermal inflammatory cell infiltration, non-edge dermal papillae. Epidermal thickness were calculated using vertical Viva Stack imaging starting immediately below the SC to the DEJ. The site starting at where the honeycomb pattern emerged and reaching down to the was measured at five sites to reach the average based on the total value. The diameters of all clearly identified loop capillaries were measured using VivaScan 7.0 Software (Lucid Inc., Rochester, NY, USA), which allowed precise measurements of the structures after calibration of the images.

Munro and Kogoj microabscesses was the main inflammatory cells within the epidermis, which is characterized by groups of round-oval-elongated, slightly refractive inflammatory cells localized at SS with RCM. The density of Munro and Kogoj microabscesses in the images of the epidermal layers from the five sites was assessed at intervals of 15 μm. The total number detected one by one was divided by five to determine the average number (number/cm^2^). Dermal inflammatory cells are seen as round-oval-elongated shaped, moderately refractive cells in the nonedge DP. For dermal inflammatory cell infiltration valuation, the inflammatory cells in the perivascular site at the DP level that were nonmobile in the video images on the most clearly visible plane were counted (number/cm^2^). The total number of cells was divided by five to determine the average number.

### Statistical analysis

Visualization of single RCM features occurrence and modification during treatment were evaluated using SPSS software (SPSS version 21.0, SPSS Inc., Chicago, IL, USA), and MedCalc (10.0.1) statistical programs were employed for all analyses. The percentage of absence or presence of every single confocal feature during the entire therapeutic follow-up was calculated. The Friedman test was applied to compare the distribution of the confocal features at different times to identify significant variations. A score was subsequently determined by analyzing the group of confocal features describing the involvement of the parakeratosis, acanthosis, inflammatory cells in epidermis and dermis, non-edge dermal papillae, vascularization in the papillary dermis. The obtained scores were then compared using the Friedman and Wilcoxon tests. The observation time was meticulously evaluated two by two using the Wilcoxon test, and the McNemar adjusted test was used for multiple comparisons. Logistic regression analysis was performed for predicting the probability of clinical relapse based on the PASI, the capillary and papillary diameters at the end of treatment as well as the percentage reduction of the PASI during therapy.

## Ethics approval and informed consent

This study was approved by the Research Ethics Committee of Wuhan No.1 Hospital and conformed to the Declaration of Helsinki. All patients provided written informed consent before inclusion.

## Results

**Figure 1:**
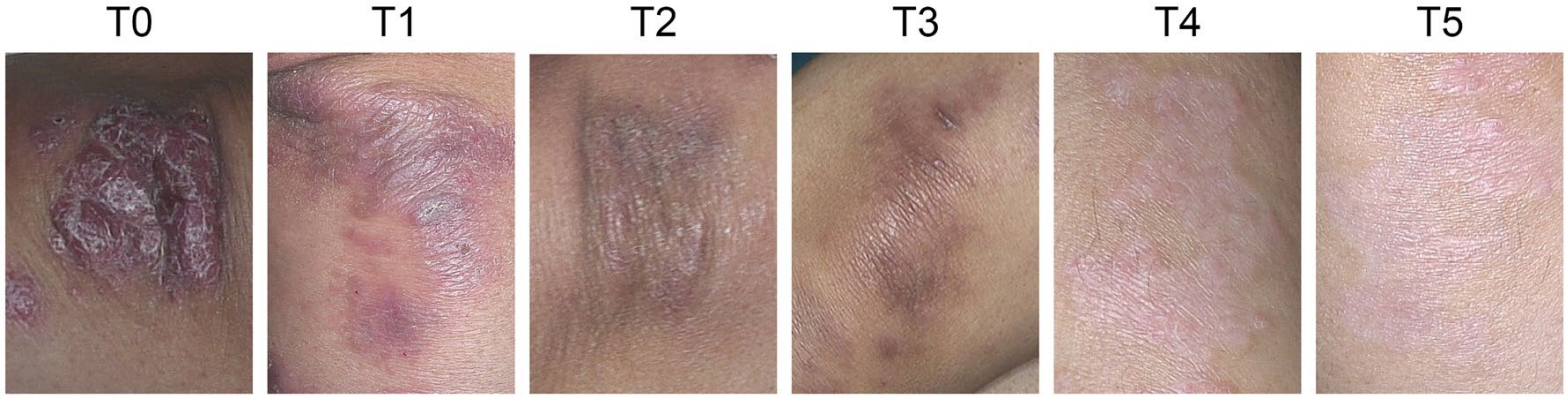
Clinical images of PP at different time points during secukinumab administration.

**Figure 2:**
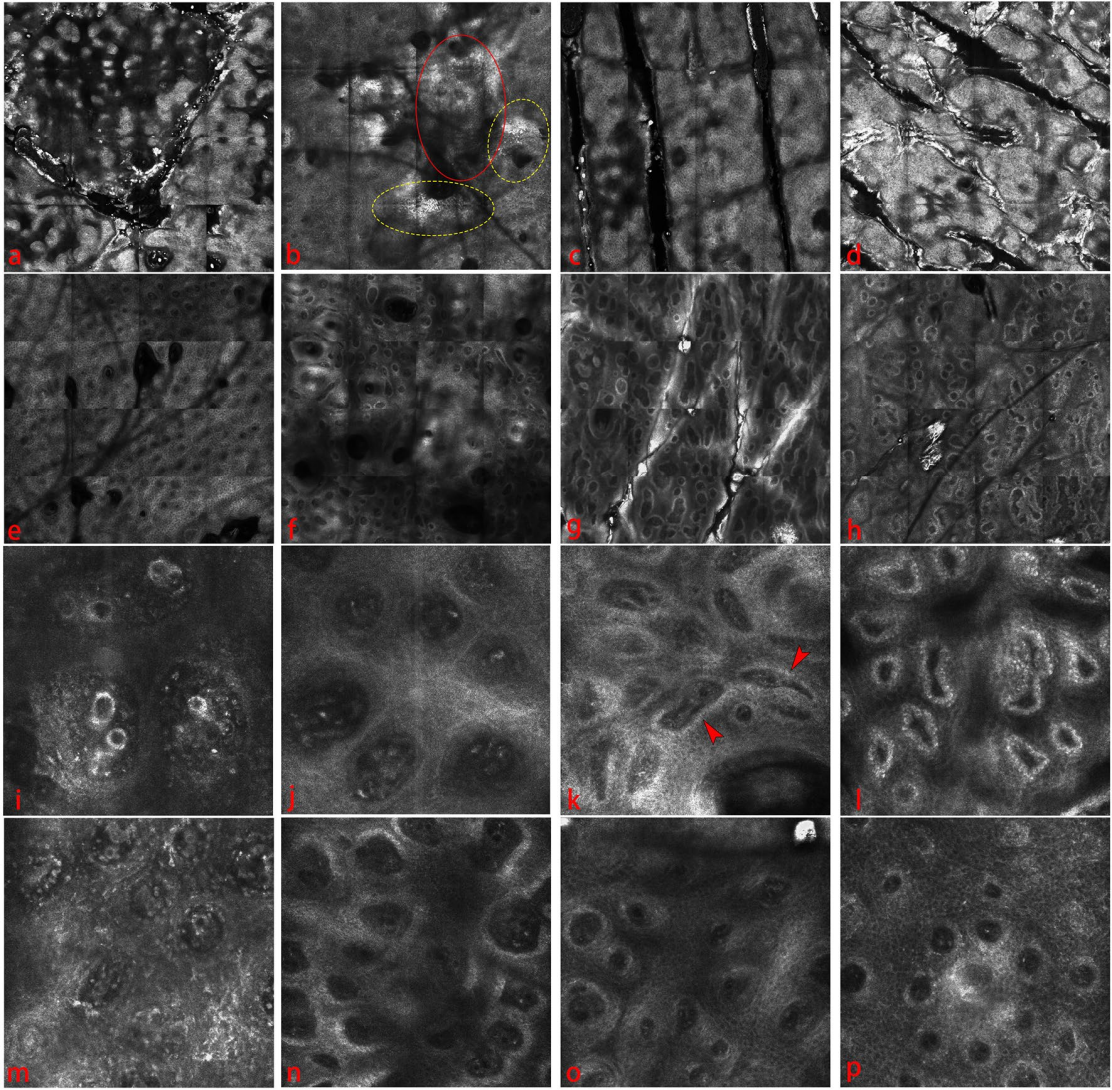
Therapeutic follow-up for PP. T0–T5: (**a**–**h**) RCM Viva Block mosaic (2 × 2 mm), (**i**–**p**) corresponding 0.5 × 0.5 mm RCM images: (**a**) RCM close-up of the papillomatosis detected at baseline. (**b**) RCM images taken at the stratum corneum and epidermis level, at baseline, areas of parakeratosis (red circle), and the presence of neutrophils, detected as bright, round to polygonal structures (yellow circle). (**c**) Recovery of the epidermis after two weeks of treatment as a sign of therapeutic response; the gradual disappearance of inflammatory cells and DP were progressively undetectable in the upper epidermal layers. (**d**) Normalization of the epidermis. The RCM image showed a normal honeycomb pattern with brighter and better-outlined keratinocyte contours and the absence of up-located DPs. (**e**) All DPs had a non-edge appearance at T0. (**f**–**h**) The DP had contours progressively, and edge DP with decreased multilocular appearance was observed in response to treatment. (**i**) Magnified window exhibiting the enlarged DP presented multilocular appearance. (**j**) The dark, dilated and tortuose canalicular structures indicated dilated vessels. (**k**) The dilated blood vessels persisted in T2–3, however, showed horizontal orientation (red arrows). (**l**) At T5, there is still a thin capillary vessel inside the edge DP that appears linear and slightly refractive. (**m**) Dermal inflammatory cells are round-oval-elongated, moderately refractive cells in the non-edge DP. (**n**–**p**) Progressive reduction in DP diameter, disappearance of inflammatory cells in the upper dermis, and appearance of bright DP rimming.

The study group comprised 14 males and 9 females. The age of the patients is in the range of 24–56 years, and the median age was 37.8 ± 11.67 years. A visible and progressive improvement in the symptoms of PP over 1 month induction therapy was observed in patients receiving secukinumab treatment (Fig. 1). The demographic and clinical characteristics of psoriasis patients treated with secukinumab is shown in Supplementary Table 1. A total of 100 untreated psoriatic plaques localized on the trunk (46), upper (34), and lower (20) extremities were assessed using RCM. The RCM criteria for psoriasis were based on the histological criteria to diagnose PP. We evaluated the RCM features of the 100 target PP lesions: parakeratosis, acanthosis, epidermal inflammatory cells, non-edge dermal papillae (DP), vascularization in the papillary dermis, and inflammatory cells in the upper dermis (Fig. 2). Two experienced RCM specialists independently analyzed all quantifiable RCM findings to ensure the reliability of the results. The parameters examined by the RCM are presented in Table 1.

**Table 1:** Confocal features and their definitions.

RCM features	Definition
Parakeratosis	The presence of nucleated cells in the stratum corneum is visualized as bright nuclei in dark corneocytes
Acanthosis (epidermal thickness)	Evaluated using VivaStack software analysis considering all cellulated layers until the total appearance of the dermal fibers
Inflammatory cells in the epidermis	The presence of bright, round-to-polygonal cells in the epidermis
Non-edge DP	Presence of dark dermal papillae more enlarged, not surrounded by bright ring of basal keratinocytes, and adjacent to each other, separated by a thin septum with a coarse fibrillary material
Vascularization in the papillary dermis	Round-to-canalicular dark structures with thin walls, inside the DP with multilocular appearance, at the level of the papillary dermis
Inflammatory cells in the dermis	The presence of bright or mildly refractive, round-to-polygonal cells around blood vessels in the dermis

This difference in the RCM results across all time points can easily be observed using RCM. RCM Vivablock of PP reveals more prominent and diffusely distributed papillomatosis with enlarged dermal papillae increased in number and density as a sign of elongation of the rete ridges and hyperkeratosis (Fig. 2a). At baseline, 32% of the lesions exhibited parakeratosis, visualized as the retention of bright nuclei in dark corneocytes (Fig. 2b). Its modification during the therapeutic follow-up was statistically significant; nearly no lesions presented parakeratosis after two weeks (T2) (Fig. 2c,d). The severity of the acanthosis was evaluated using Vertical Viva Stack software analysis. The site starting at where the honeycomb pattern emerged and reaching down to the DEJ was measured at five sites to reach the average based on the total value (μm). Acanthosis was observed in all lesionsat baseline, the mean thickness of epidermis was 94.47 ± 21.27 µm. Although the thickness of the stratum corneum was reduced at T2 (78.46 ± 13.17 µm), the difference was insignificant between T4 and T5 (Table 2). Parakeratosis and acanthosis summarize the epidermal involvement in PP and illustrate the treatment’s impact on restoring normal epidermal proliferation.

**Table 2:** Friedman test for the evaluation of the statistical significance of the scores related to the groups of confocal criteria defined in Fig. 3 in relation to the response to treatment. Wilcoxon test for pair comparison in relation to the timing of observation.

	Parakeratosis	Inflammatory cells in epidermis	Non-edge dermal papillae	Inflammatory cells in dermis	Vascularization in the papillary dermis	Achantosis
Friedman test (χ2)	*P* = 0.0004 (22.74)	*P* < 0.0001 (35.29)	*P* < 0.0001 (45.62)	*P* < 0.0001 (48.82)	*P* < 0.0001 (189.0)	*P* < 0.0001 (151.8)
Wilcoxon test						
T1 vs. T0	*P* = 0.0469	*P* = 0.0078	*P* = 0.0039	*P* = 0.0020	*P* < 0.0001	*P* = 0.0004
T2 vs. T0	*P* = 0.0313	*P* = 0.0039	*P* = 0.0020	*P* = 0.0020	*P* < 0.0001	*P* < 0.0001
T3 vs. T0	*P* = 0.0313	*P* = 0.0039	*P* = 0.0020	*P* = 0.0020	*P* < 0.0001	*P* < 0.0001
T4 vs. T0	*P* = 0.0313	*P* = 0.0039	*P* = 0.0020	*P* = 0.0020	*P* < 0.0001	*P* < 0.0001
T5 vs. T0	*P* = 0.0313	*P* = 0.0039	*P* = 0.0020	*P* = 0.0020	*P* < 0.0001	*P* < 0.0001
T2 vs. T1	*P* > 0.999	*P* = 0.6250	*P* = 0.0078	*P* = 0.0039	*P* < 0.0001	*P* = 0.0005
T3 vs. T2	*P* > 0.999	*P* = 0.5000	*P* = 0.0156	*P* = 0.0039	*P* = 0.0004	*P* < 0.0001
T4 vs. T3	NA*	NA	*P* = 0.5000	*P* = 0.0078	*P* = 0.0183	*P* = 0.0010
T5 vs. T4	NA	NA	*P* = 0.5000	*P* = 0.500	*P* = 0.1185	*P* = 0.9248

*NA: not available.

Neutrophils play a well-documented role in the pathophysiology of psoriasis. Pathognomonic neutrophil collections are frequently identified as clusters of highly refractile round to polygonal cells at the stratum spinosum and stratum corneum, where they form micropustules of Kogoj and microabscesses of Munro, respectively. Highly refractile nucleated cells, consistent with neutrophils, were found in 45% of the lesions at T0 (Fig. 2b). During treatment, the considerable reduction in epidermal focal microabscesses could be easily distinguished morphologically by RCM.

In psoriasis lesions, RCM virtual horizontal sections at the level of the DEJ exhibited numerous conspicuous DP with a multilocular appearance. One of the main RCM features observed at the DEJ was the presence of a non-edge DP. Unlike the DP of healthy skin, they were well-defined, more enlarged, adjacent to each other, not surrounded by any bright ring of basal cells. Instead, they were separated by a thin, slightly refractile septum (Fig. 2e). DP in psoriasis may be due to the inhibitory effect of interleukin-17A on melanogenesis. The recovery of DEJ bright rimming, considered as a signal of melanocyte re-activation, has been independently examined. A significant number of lesions (83%) exhibited non-edge DP at baseline; however, DP rimming became statistically significant during treatment between T0 and T1 and T0 and T2 (Fig. 2f–h) (Table 2). A reasonably robust and positive correlation was observed between the variation in DP rimming and the progression in the PASI score from the baseline to T5 (Table 3).

**Table 3:** Correlation analysis of confocal criteria defined in Fig. 3 and PASI score.

	Parakeratosis	Inflammatory cells in epidermis	Non-edge dermal papillae	Inflammatorycells in dermis	Vascularization in the papillary dermis	Achantosis
Correlation	0.422	0.548	0.796	0.829	0.825	0.793
Sig. (2-tailed)	*P* = 0.001	*P* < 0.001	*P* < 0.001	*P* < 0.001	*P* < 0.001	*P* < 0.001

Distributions are represented in the main diagonal and correlation coefficients in corresponding graphs. Data is represented together with a 95% density ellipse and a least squares regression line with a 95% confidence region.

**Figure 3:**
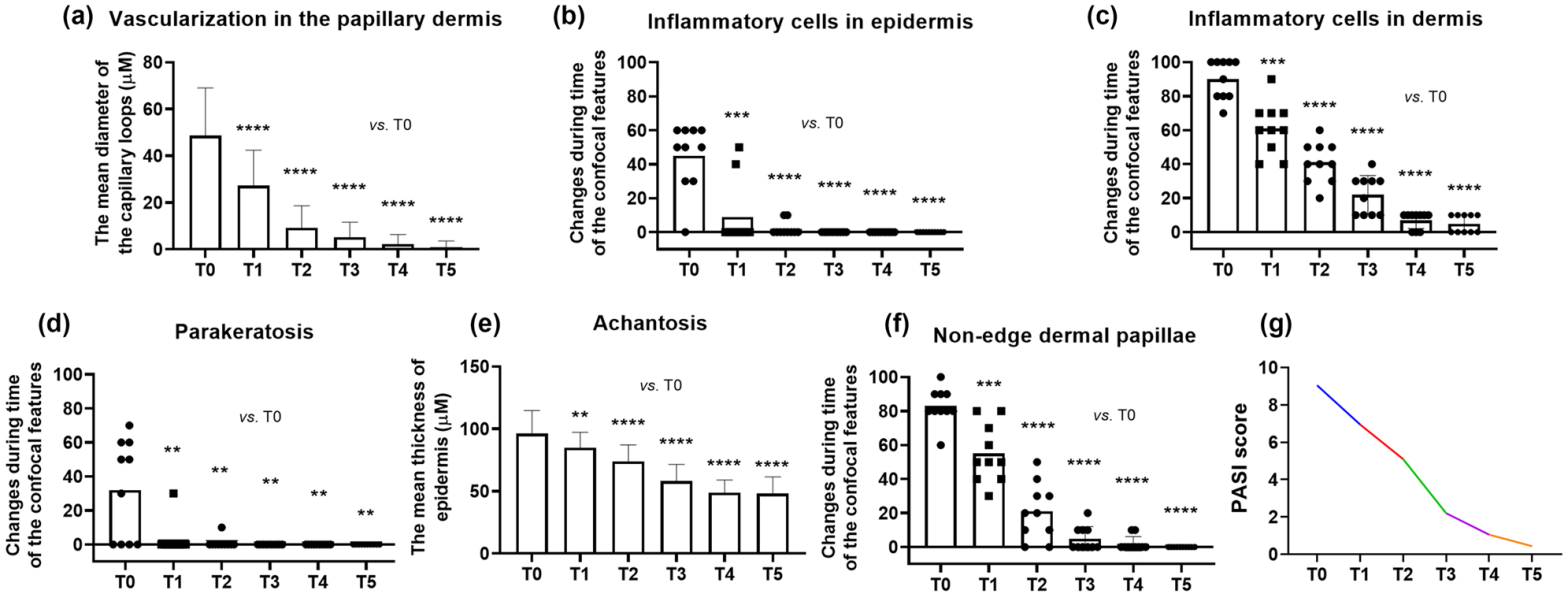
Comparison the confocal features: vascularization in the papillary dermis (**a**), inflammatory cells in the epidermis (**b**) and upper dermis (**c**), parakeratosis (**d**), acanthosis (**e**), non-edge dermal papillae (**f**) and the and median PASI score (**g**) between the baseline and the different times during Secukinumab treatment. Values are expressed as the mean ± standard deviation. *P < 0.05, **P < 0.01, ***P < 0.001, ****P < 0.0001.

PP pathophysiology includes vascularization in the papillary dermis, which was successfully identified on RCM virtual sections as enlarged DP presented multilocular appearance (Fig. 2i). The capillary vessels to become dilated, describing multiple loops in their trajectory, and visible as prominent dark canalicular structures, dilated, and tortuose, filling the papillary dermis throughout the DP rings in 100% of the lesions (Fig. 2j). Micromorphological analysis using the VivaScan 7.0 Software revealed that the mean values of the capillary loop diameter were significantly higher in PP lesions compared to normal skin. Figure 3 summarizes the parameter changes in the capillary loops determined by RCM at all time points. The mean diameter of the capillary loops was 46.59 ± 19.26 µm at baseline in the lesions at T0. Conversely, the dilated and tortuose canalicular structures were not detectable in the patient’s normal-appearing skin (at least 5 cm from the lesion site). The diameter of blood vessels was considerably reduced at T1 (36.36 ± 15.9 µm) and T2 (22.86 ± 4.88 µm) compared with baseline. The difference was statistically significant (*P* < 0.0001).

Despite the presence of a weak but positive association between vascularization in the DP and the PASI score and minimal papillary blood flow at T3, dilated blood vessels in the DP persisted until T5 (Fig. 2l). A transition in the orientation of the blood vessels was also observed during the treatment. At baseline, PP lesions exhibited vertically oriented blood vessels filling the DP because of up-migrated DP. Enlarged DP presented multilocular appearance, canalicular or round dark spaces represent vessels that run perpendicular to the surface. During treatment, the orientation of the blood vessels was converted; dilated blood vessels (started at T2) showed horizontal orientation and linear formations in the upper dermis (Fig. 2k, l), corresponding to a parallel (en face) orientation to the skin surface.

The rapid movement of numerous prominent, highly refractive polymorphonuclear leukocytes in dark capillary lumen, and presence of mildly refractive monocytes surrounding the dilated vessels in the papillary dermis was identified in all lesions (Fig. 2m). Conventional histology has confirmed that these cells correspond to perivascular inflammatory cell infiltration. A significant decrease in dermal inflammatory cells was observed because of the treatment response compared with the baseline. Dermal inflammatory cells persisted in T5 (Fig. 2n–p), but the reduction was statistically significant.

The PASI score showed progressive global clinical improvement in all patients enrolled in the research. These RCM criteria were compared with the clinical scale evaluation and PASI scores, and the outcomes were statistically significant during follow-up (Table 2). Treatment outcome scores and changes of the confocal features categorized by gender during treatment with Secukinumab is shown in Supplementary Fig. 1.

## Discussion

Biological agents exemplify targeted molecular therapy, demonstrating good efficacy and improving the quality of life of patients with a low risk of toxic effects^14^. Various methods have been implemented to evaluate the safety and effectiveness of innovative biological therapies for psoriasis^14^. These methodologies can be evaluated based on changes in severity scores during and after treatment, including BSA and PASI. However, the existing psoriasis scoring systems possess several limitations because the interpretation may be subjective with interobserver variation. These restrictions can be resolved by performing paired skin biopsies before and after therapy. Nonetheless, skin biopsy is an invasive technique and represents only a tiny sample of the lesion, which potentially results in sample error. Therefore, biopsy is certainly an imperfect way to dynamically monitor intracutaneous alterations and the histological effects of secukinumab therapy for psoriasis. The RCM analysis of PP can be used to overcome the constraints associated with biopsies. This study revealed the effectiveness of RCM for detailed monitoring of microscopic changes and therapeutic follow-up of PP during treatment with secukinumab.

Secukinumab is a recombinant, high-affinity, fully human immunoglobulin G1κ monoclonal antibody that selectively binds and neutralizes interleukin-17A, which stimulates keratinocytes to secrete chemokines and other proinflammatory mediators that recruit additional inflammatory cells, potentially acting as a master cytokine in psoriasis pathogenesis^15^. We evaluated the microscopic signs of inflammation during the therapeutic follow-up. After four weeks of treatment, the anti-inflammatory activity of secukinumab diminished epidermal and superficial dermal inflammatory infiltration. The early disappearance of the micropustules of Kogoj and microabscesses of Munro at the stratum spinosum and stratum corneum levels was immediately followed by the disappearance of parakeratosis as a sign of progressively normalizing keratinocyte maturation. This characteristic is associated with secukinumab’s renowned early anti-inflammatory activity. Similarly, acanthosis and epidermal and superficial dermal inflammatory cell infiltration showed a substantial and drastic reduction at T2–T3. Moreover, the early recovery of the bright rims surrounding the DP was identified on RCM at T1 with statistical significance. This confocal feature, corresponding to the early re-appearance of DEJ pigmentation of keratinocytes, may indicate an early effect of secukinumab on melanocyte activity.

All PP lesions considered in the study showed clinically assessable improvement after four weeks of secukinumab standard protocol therapy, with a few residual post-inflammatory macules. Substantial clinical improvement and a nearly normal DP configuration were observed on RCM. However, some characteristics of psoriasis can still be observed through RCM. For instance, the infiltration of inflammatory cells into the epidermis and the diameter of dilated vessels decreased quickly at T0–T1 and T1–T2; however, this change was not statistically significant between T4 and T5. Dilated vessels in DP continued in T5 and was still linked with a few remaining inflammatory cells, which characterize the inflammatory status of PP lesions. The RCM highlighted the need for a longer period for thorough resolution of subclinical alterations despite complete clinical response. The RCM may offer novel perspectives on the subclinical evaluation of special-site responses to biological therapy.

Multiple authors studying in vivo examination of psoriatic skin supported the hypothesis that microvascular alteration is crucial in developing and maintaining psoriatic skin lesions. Furthermore, there is an assumption of a correlation between the modification of skin vessels and the activation of basal keratinocytes. Vascularization in PP occurs earlier than the surface manifestations of the skin and even before the histological observation of epidermal hyperplasia^16^. This research revealed the capability of RCM to ascertain the parameters of dermal vessels. Compared with the skin of healthy subjects, capillaries examined in lesions of PP exhibit higher mean values of micromorphological parameters (area, perimeter). Moreover, the number of capillary sections is higher for each papilla. In this study, the clinical improvement was associated with a substantial reduction in the capillary loop diameter, and the normalization of the papillary structure of plaque skin. Additionally, this is the first study demonstrating that the vascular orientation and pattern of PP lesions, may undergo a transition during treatment. At baseline, a peculiar vertically oriented blood vessels filled the DP; however, after secukinumab therapy, horizontal orientation of dilated blood vessels in the upper dermis was observed. This transformation is visible at the horizontal approach of RCM as the disappearance of the up-located DP and the progressive thickening of the inter-papillary spaces. When comparing the findings of logistic regression analysis with PASI, we may assume that there is a better tendency for skin vessel normalization in predicting the course of psoriasis after treatment and, more generally, in gauging the efficacy of therapies. The percentile reduction in PASI showed a similar predictor status of recurrence, as capillary diameters and orientation after treatment.

Our findings indicated that the therapeutic response to secukinumab is also affected by the anatomical localization of the lesions, and clinical assessment failed to identify the corresponding reduction in the erythema in plaques located on the legs. However, the clinical clearance status of erythema and normalization of the microvascular alterations in RCM is better in lesions on the trunk, thighs, and upper arms. This apparent contradiction may be explained by the evidence noted on RCM, revealing that dermal vessels continued until T5 in lesions located on the legs, which is recognized as the main microscopic factor determining the erythema characteristic of PP. One could hypothesize that microcirculation impairment frequently results in elevated hydrostatic pressure in the leg anatomical region. The delay of normalization of the microvascular changes in leg localization could result from increased hydrostatic pressure-induced inflammatory activity. It is widely recognized that increased hydrostatic pressure causes an increase in vessel permeability, thereby enabling fibrinogen to escape into the pericapillary tissue. Consequently, a fibrin cuff is formed around dermal capillaries, leading to tissue hypoxia, cell damage, and release of inflammatory mediators^17^. We theoretically speculated that these factors may decrease the anti-inflammatory effects of secukinumab therapy and additional factors may also contribute to the less efficacy of the drug on the lower limbs.

In conclusion, the subclinical characteristics of psoriasis and the timing of the normalization of the histopathological characteristics during treatment can be identified without biopsy through RCM. Monitoring histological parameters during therapy provides significant insights into the therapeutic response, the mechanism of action of therapeutic agents, and the pathological mechanisms involved in developing cutaneous lesions.

### Supplementary Information

Supplementary Figure 1.

Supplementary Table 1.

## Data Availability

The data underlying this article are available in the article and in its online supplementary material.
