# Supplementary figures and images for: Reflectance confocal microscopy for plaque psoriasis therapeutic follow-up during an anti-interleukin-17A monoclonal antibody: an observational study
Source: Sci Rep. 2024 Jul 2;14:15121. doi: 10.1038/s41598-024-65902-8 (PMC11219718; doi:10.1038/s41598-024-65902-8)

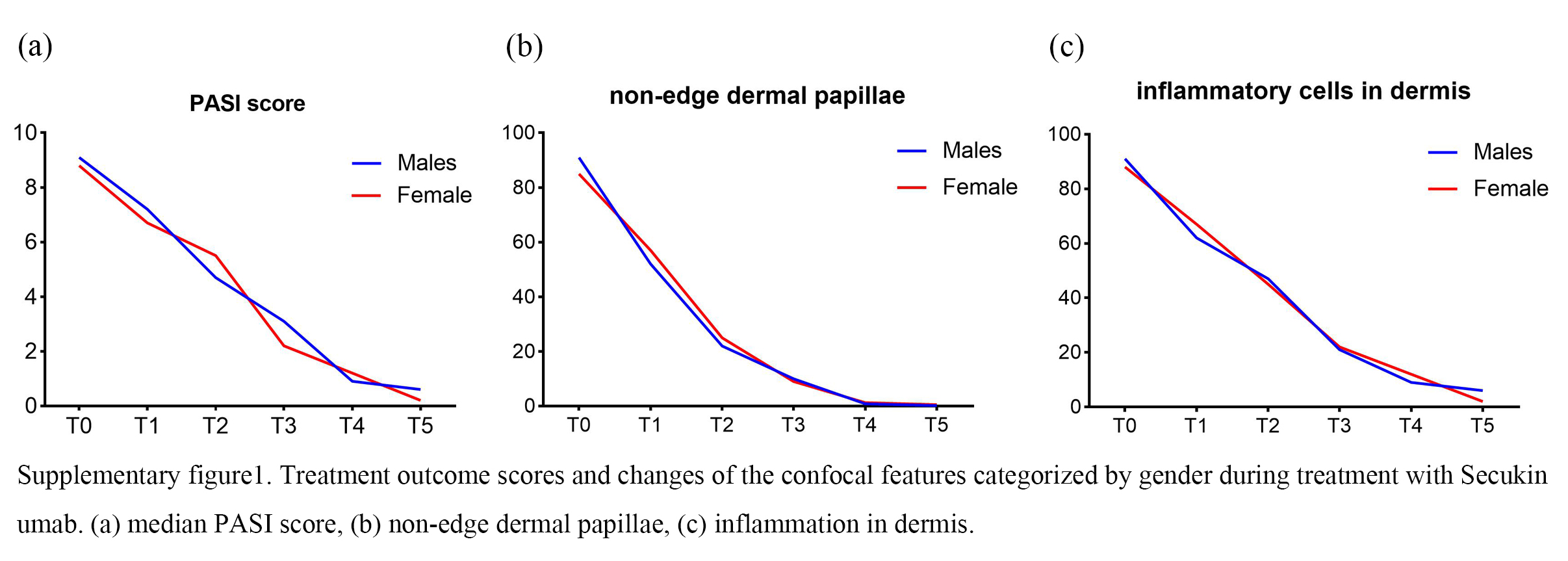

Supplement: Supplementary file 1 — Supplementary Figure 1. [file 41598_2024_65902_MOESM1_ESM.jpg]
