# Supplementary material for: Reflectance confocal microscopy for plaque psoriasis therapeutic follow-up during an anti-interleukin-17A monoclonal antibody: an observational study
Source: Sci Rep. 2024 Jul 2;14:15121. doi: 10.1038/s41598-024-65902-8 (PMC11219718; doi:10.1038/s41598-024-65902-8)
Supplement: Supplementary file 2 — Supplementary Table 1. [file 41598_2024_65902_MOESM2_ESM.docx]

| Table. Demographic and clinical characteristics of psoriasis patients treated with secukinumab (n= 23). | | |
| --- | --- | --- |
| Age | Range | 24 - 56 |
|  | Mean ± SD | 37.8 ± 11.67 |
| Sex (N, %) | Males | 14 (60.87%) |
|  | Female | 9 (39.13%) |
| Race | Asian | 23 (100%) |
| Fitzpatrick skin phototype | Ⅰ | 0 |
|  | Ⅱ | 1 (4.35%) |
|  | Ⅲ | 9 (39.13%) |
|  | Ⅳ | 12 (52.17%) |
|  | Ⅴ | 1 (4.35%) |
|  | Ⅵ | 0 |
| Types of psoriasis | Psoriasis vulgaris | 23 (100%) |
| PASI Score at T0 | Median | 9.1 |
|  | Range (min-max) | 4.2 - 14.6 |
| PASI Score at T1 | Median | 6.8 |
|  | Range (min-max) | 2.8 - 11.9 |
| PASI Score at T2 | Median | 5 |
|  | Range (min-max) | 2.1 - 6.3 |
| PASI Score at T3 | Median | 2.2 |
|  | Range (min-max) | 1.2 - 5.7 |
| PASI Score at T4 | Median | 1.1 |
|  | Range (min-max) | 0 - 3.3 |
| PASI Score at T5 | Median | 0.4 |
|  | Range (min-max) | 0 - 1.2 |
